# Identifying barriers and coping strategies for pre-exposure prophylaxis disclosure: experiences of Ugandan adolescent girls engaged in transactional sex—a qualitative study

**DOI:** 10.3389/fgwh.2025.1517448

**Published:** 2025-03-20

**Authors:** Simon Mwima, Laura M. Bogart, Steela Neema, Richard Komo, Stephen Obbo

**Affiliations:** ^1^ Department of Social Work, University of Illinois Urbana Champagne, Champaign, IL, United States; ^2^ Bukedi Prevention Institute, Mbale, Uganda; ^3^ Department of Behavioral Science, RAND, Los Angeles, CA, United States; ^4^ Department of Behavioral Science, Charles R. Drew University of Medicine and Science, Los Angeles, CA, United States; ^5^ Makerere University School of Sociology, Kampala, Uganda; ^6^ Department of Social Work, Mbale Regional Referral Hospital, Mbale, Uganda; ^7^ Department of Clinical Medicine, Mbale Regional Referral Hospital, Mbale, Uganda

**Keywords:** pre-exposure prophylaxis (or PrEP), PrEP disclosure, adolescent girls and young women (AGYW), transactional sex, biomedical HIV prevention, Uganda (sub-Saharan Africa)

## Abstract

**Introduction:**

The disclosure of oral pre-exposure prophylaxis (PrEP) use among adolescent girls engaged in transactional sex in Uganda is a complex process shaped by stigma, fear of judgment, misconceptions, and the threat of violence. This qualitative study explores the strategies these adolescents use to navigate these challenges, drawing on resilience theory and asset-based approaches.

**Methods:**

Between April 2018 and May 2019, cross-sectional semi-structured interviews were conducted with Ugandan adolescent girls aged 18–24 engaged in transactional sex to explore their experiences of PrEP disclosure. Data were analyzed using a thematic network analysis approach, focusing on how participants managed the social and psychological barriers to disclosure.

**Results:**

The study found that stigma, misconceptions about PrEP, and fear of judgment or violence from clients and families were significant barriers to disclosure. However, adolescent girls employed multiple strategies to navigate these challenges. These included relying on peer support, selective disclosure to trusted individuals, and drawing on personal strength and resilience. Support from healthcare providers and access to youth-friendly health services further helped participants manage the risks associated with PrEP disclosure. Many participants used a combination of these strategies, adapting their approach to different social contexts.

**Conclusion:**

This study highlights the complex strategies adolescent girls engaged in transactional sex in Uganda use to disclose PrEP use amidst significant barriers. The findings emphasize the need for targeted interventions that focus on strengthening peer support, enhancing the role of healthcare providers, and creating safe spaces for disclosure. By integrating these strategies into PrEP delivery models, public health efforts can empower these vulnerable populations to disclose and adhere to PrEP more confidently, improving HIV prevention outcomes.

## Introduction

Human Immunodeficiency Virus (HIV) remains a global public health and social justice challenge, particularly in Sub-Saharan Africa, where vulnerable populations like adolescent girls and young women (AGYW) engaged in transactional sex face a heightened risk of infection. In 2023, an estimated 1.7 million new HIV infections occurred worldwide, with sub-Saharan Africa accounting for over 70% of these cases (1). In Uganda, HIV incidence remains high, with an estimated 50,000 new infections occurring annually (2). Key populations, such as adolescent girls engaged in transactional sex, are disproportionately affected by HIV, with prevalence rates in this group significantly higher than the national average of 5.5% in Uganda (2). Studies have shown that adolescent girls in this population face an increased HIV risk, with rates ranging between 20% and 40% due to factors such as limited access to healthcare, intersectionality of stigmas, and economic dependence on sex work (3–5). Given the heightened vulnerability, there is an urgent need to expand biomedical HIV prevention efforts among key populations.

Daily oral pre-exposure prophylaxis (PrEP) is a highly effective biomedical intervention that uses antiretroviral drugs to prevent HIV transmission, particularly through sexual intercourse (6, 7). It is especially critical for high-risk populations like AGYW engaged in transactional sex, providing a crucial tool for HIV prevention (8, 9). When taken consistently, PrEP can reduce the risk of HIV infection by over 90% (8, 10). However, despite its proven efficacy, real-world uptake and adherence to PrEP are often challenged by significant barriers, especially around the issue of PrEP disclosure. PrEP disclosure—informing partners, peers, family, or healthcare providers about PrEP use—is essential for adherence and the broader public health impact of the intervention (11). While disclosure can foster social support and help mitigate stigma, many AGYW engaged in transactional sex face substantial barriers to openly discussing their PrEP use.

Barriers to PrEP disclosure among AGYW engaged in transactional sex in Uganda are diverse and multifaceted and exist across multiple levels of the social-ecological model. At the individual level, personal barriers including fear of judgment, stigma and potential for violence significantly hinder PrEP disclosure (12–14). Many AGYW fear being perceived as promiscuous or living with HIV due to due to widespread misconceptions about PrEP's purpose believing that PrEP is intended only for individuals already living with HIV (15). These fears are compounded by the socio-economic realities of transactional sex, where disclosure may result in economic repercussions or violence from clients who misunderstand the role of PrEP (3). At the interpersonal level**,** relationships with clients, family, and peers play a critical role. AGYW face a double stigma: first, for their involvement in sex work, and second, for PrEP use, which is often misunderstood as a sign of HIV positivity (16–18). This dual stigmatization leads to social rejection and isolation from key social networks, making open disclosure even more challenging. However, studies have shown that supportive interpersonal relationships—with family, peers, or partners—can facilitate PrEP disclosure and improve adherence (17, 19). At the community level**,** pervasive sociocultural norms and stigma surrounding both sex work and HIV prevention create hostile environments for PrEP disclosure. Misconceptions and deeply entrenched biases within communities fuel discriminatory practices, further marginalizing AGYW who use PrEP (19, 20). Community-level stigma can result in social isolation and even gender-based violence following PrEP disclosure, which negatively affects adherence and continuation (3, 21, 22). At the organizational level, limited access to youth-friendly healthcare services creates additional barriers to PrEP disclosure. Many healthcare providers lack the training or willingness to offer non-judgmental, supportive care, and healthcare systems often fail to provide safe spaces for AGYW to discuss their PrEP use openly (23, 24). Healthcare workers' negative attitudes and a lack of culturally sensitive services further discourage AGYW from seeking guidance or support related to PrEP disclosure (25). At the structural and policy level**,** broader systemic issues such as healthcare infrastructure limitations and inadequate policies that fail to protect AGYW from stigma and violence play a critical role. Structural barriers include insufficient legal protections for sex workers and the absence of policies that promote equitable healthcare access for marginalized groups. The lack of comprehensive public health campaigns to correct misconceptions about PrEP exacerbates stigma at all levels (20, 23, 24). Understanding these factors multi-level factors is crucial for developing targeted interventions that address the complex barriers to PrEP disclosure.

To date, most studies have taken a deficit-based approach, focusing primarily on the barriers that hinder PrEP disclosure (26–29). Few studies have explored the strategies that these girls use to navigate these barriers, especially in the Ugandan context. This gap underscores the need for a strengths-based approach to understand better how AGYW engaged in transactional sex can be empowered to disclose their PrEP use while maintaining their safety and well-being (17, 30, 31). This study seeks to address this gap by exploring the lived experiences of AGYW engaged in transactional sex in Uganda, focusing on how they overcome the social, psychological, and structural barriers to PrEP disclosure. By examining these experiences through the strength-based frameworks, we aim to uncover strategies that can inform future interventions designed to improve PrEP disclosure among this vulnerable population.

## Theoretical frameworks

### Social-ecological model

The Social-Ecological Model (SEM) serves as the guiding framework for understanding the complex and multi-level barriers to PrEP disclosure among AGYW engaged in transactional sex in Uganda. The SEM highlights how individual behaviors, such as disclosing PrEP use, are shaped by interactions across various levels of influence. At the individual level, personal factors such as fear of judgment, misconceptions about PrEP, and concerns about potential post-disclosure violence contribute to reluctance to disclose. Moving outward, the interpersonal level focuses on relationships with peers, family, and clients, where AGYW may face rejection, stigma, or economic consequences, particularly from clients who misunderstand PrEP's purpose. The community level addresses the broader sociocultural norms and collective attitudes that perpetuate stigma against both PrEP use and sex work, reinforcing non-disclosure. At the organizational level, barriers arise from healthcare environments that lack youth-friendly services and are marked by healthcare workers' negative attitudes, making it difficult for AGYW to seek support for safe disclosure. Finally, the structural and policy level includes systemic factors such as insufficient public health education to dispel myths about PrEP, and gaps in healthcare access that further complicate disclosure. By applying the SEM, this study captures the layered and interconnected barriers that AGYW face, providing a foundation for developing multi-level interventions aimed at facilitating safer and more supportive environments for PrEP disclosure.

### Resilience, positive deviance, and asset-based approaches

Resilience, positive deviance, and asset-based approaches offer valuable perspectives for understanding how adolescent girls engaged in transactional sex in Uganda navigate the complexities of disclosing PrEP use. Resilience in this context refers to the ability to positively adapt in the face of adversity, such as the intersectionality of stigma (HIV, PrEP, and sex work) and discrimination linked to both PrEP disclosure and transactional sex (32). Resilience involves two key components: positive adaptation in the face of risk and a dynamic process of recovery (32). Fergus and Zimmerman (33) highlight that in resilience frameworks, chronic stress related to HIV prevention and adherence is a significant risk factor (33). In this study, resilience reflects how adolescent girls manage and recover from the intersectionality of stigmas and challenges tied to PrEP use in a highly stigmatizing Ugandan environment.

Although the application of resilience models to PrEP disclosure is still emerging, this study builds on recent work showing the value of resilience in HIV prevention among vulnerable populations. For example, in a study on young women in South Africa and Zimbabwe, social support from family and healthcare providers was found to increase successful PrEP disclosure (34). By applying a resilience lens, we explore how adolescent girls in Uganda overcome personal and societal challenges, emphasizing how they adapt in a context of high stigma and discrimination.

In addition to resilience, this study incorporates the Positive Deviance (PD) Approach. PD is a problem-solving framework that focuses on identifying uncommon but successful behaviors that certain individuals or groups use to overcome challenges despite having the same resources as their peers (35). In this study, positive deviance helps us understand how certain adolescent girls adopt adaptive behaviors, such as selective disclosure or seeking peer support, to successfully manage PrEP disclosure and continuation. A 2018 qualitative study found that vulnerable youth who sought support from trusted community members experienced better health outcomes in HIV prevention, despite facing significant risks (36). By identifying and promoting such behaviors, we can inform interventions that encourage similar adaptive strategies, allowing more adolescent girls to disclose PrEP use successfully. This approach offers insight into how girls shape their environments to promote safer disclosure practices.

Finally, the Asset-Based Approach focuses on identifying and leveraging community resources, such as youth-friendly health facilities, informal social groups, and healthcare workers, to enhance HIV prevention efforts (58). Asset-based models have been proposed as a way to amplify existing support networks in public health interventions, particularly in low-resource settings. For adolescent girls engaged in transactional sex, peer-led community-based organizations, and social support systems may offer essential resources for overcoming PrEP-related stigma and discrimination. Although the application of asset-based approaches in HIV prevention is still limited in developing contexts, integrating these models into PrEP interventions could significantly enhance disclosure and other outcomes such as adherence and continuation (37, 38).

Together, resilience, positive deviance, and asset-based approaches offer complementary frameworks for understanding how AGYW engaged in transactional sex in Uganda manages the barriers to PrEP disclosure. Combining these models allows for a holistic perspective beyond deficit-based approaches, highlighting the adaptive strategies and community resources that girls leverage to disclose PrEP use. By integrating these approaches, we can identify successful behaviors and community assets that promote PrEP disclosure, ultimately informing more effective interventions that build on the strengths already present in these communities (39). This combined approach shifts the focus from what hinders disclosure to what enables it, offering a more empowering narrative for adolescent girls navigating the complexities of PrEP use in Uganda.

## Methods

### Study context

This study utilized data from the Most At-Risk Populations Initiative (MARPI) clinic, located within the Mulago National Referral Hospital complex in Kampala, Uganda. The MARPI clinic was established by Uganda's Ministry of Health (MoH) in 2008 to provide specialized services to key populations at higher risk of HIV infection, including adolescent girls engaged in transactional sex. It was the first public health facility in Uganda to offer PrEP services outside of a trial setting, and by the time of data collection, the clinic was providing PrEP to over 1,500 key population clients (40). The clinic operated a daily facility-based PrEP service that offered clients initiation and continuation support. Adolescents on PrEP were scheduled for monthly follow-up appointments initially and, upon stabilization, received three-month refills. Throughout these clinic visits, disclosure and adherence were routinely evaluated. MARPI clinic staff, a Ugandan nurse-counselor, trained in youth-friendly and non-judgmental approaches, were crucial in supporting and guiding these adolescents, helping them navigate the social and emotional challenges surrounding PrEP use disclosure and sex work.

### Recruitment

Participants were recruited on PrEP clinic days at MARPI. The eligibility criteria were as follows: (1) aged between 18 and 24 years, (2) having been on PrEP for at least 6 months, (3) having exchanged sex for money or goods in the last month, and (4) providing informed consent. Participants were excluded from the study if they had a severe illness or any contraindications to oral PrEP, such as confirmed hepatitis B or kidney failure (41, 42). Recruitment was conducted over a 3-week period by a healthcare provider who identified and screened 18 eligible participants who had physically come to the clinic for routine follow-up and referred them to two trained research assistants; of those screened, 14 participants were included in the study. The research assistants explained the study's purpose and screened participants for eligibility using a recruitment script. Informed consent was obtained, and participants were compensated for their time and transport costs. This study adheres to the Consolidated Criteria for Reporting Qualitative Research (COREQ) Checklist to ensure transparency and rigor in reporting qualitative methods and findings (Supplementary File 1: COREQ checklist).

## Data Collection

### Participants

A total of 14 AGYW engaged in transactional sex were purposively selected and participated in semi-structured interviews. The sample reached saturation as no new themes emerged, making it sufficient to address the research questions. Participants had disclosed PrEP use. Adolescents aged 18–24 who had successfully disclosed PrEP use were invited to participate. Informed consent was obtained from all participants before the interviews were conducted.

The interviews were conducted by two experienced Ugandan research assistants who audio-recorded the interviews and took field notes: a male master's student and a female nurse-counselor both deeply committed to working with marginalized populations. The male assistant, with over 6 years of experience as a medical social worker in Uganda's healthcare system, brought valuable insights into patient care and community health dynamics. The nurse-counselor, holding a Bachelor of Science in Nursing (BSN) and a degree in Counseling, contributed more than 7 years of experience providing psychosocial support to vulnerable groups. Their combined expertise fostered a supportive environment that encouraged trust and open dialogue during the interviews, which were held in a private room within the MARPI clinic to ensure confidentiality and lasted approximately 1 h. A semi-structured interview guide was used to explore participants' experiences with PrEP disclosure, including barriers they faced, the social, psychological, and instructional strategies they used to navigate these challenges, and their interactions with healthcare providers. All interviews were conducted in English and were audio-recorded with participant consent. To create rapport, interactive icebreakers and education conversations on PrEP and sexual health were provided, allowing participants to feel comfortable, informed, and respected before sharing their experiences. During the interviews, participants used pseudonyms or remained anonymous if they preferred. Participants received 20,000 Ugandan Shillings (approximately $5 USD) to compensate for their time and transport.

### Data analysis

Audio recordings were transcribed verbatim and organized using Dedoose (version 7.0.23) for data management. A total of 1,425 passages were extracted from the transcripts, and thematic network analysis (43) was employed to identify key themes related to barriers to PrEP disclosure and the strategies used by participants to navigate these challenges. This approach allowed for inductive exploration of emergent themes and deductive examination of theoretical constructs, such as resilience, positive variation, and asset-based frameworks. The analysis followed a four-stage Thematic Network Analysis (TNA) approach. In the first stage, initial open codes were generated. Coding was conducted where transcripts were reviewed, and key phrases were generated from the data to capture meaningful concepts related to barriers to PrEP disclosure and the strategies participants used to disclose PrEP use. In the second stage, basic themes were identified, clustering similar issues discussed based on participants' experiences (e.g., stigma, fear of violence, lack of privacy, and social support). In the third, the basic themes were organized into themes, which grouped related ideas into the broader analytical and final stages categories. Finally, in the fourth stage, these global themes were reviewed and refined by linking organizing themes into overarching concepts that provided deeper insights into the research questions. This paper reports emerging basic themes that speak to two pathways (organizing themes) to barriers and strategies that facilitate PrEP disclosure (global theme). Table 1 outlines the thematic network and structure guiding the results section.

**Table 1:** Thematic network analysis (TNA) for barriers and strategies for PrEP disclosure among adolescents and young women engaged in transactional sex in Uganda.

Basic theme	Issues discussed in codes	Organizing theme	Global theme
Confusion between PrEP and ARVs and the association of PrEP with sex work	Belief that PrEP is HIV treatment, community misinformation about PrEP, assumptions of being HIV positive, false rumors and gossip, avoidance by family and friends, exclusion from social and professional spaces, discrimination due to HIV stigma, belief that PrEP is for sex workers, moral judgment from the community	Misinformation and stigma	Barriers to PrEP disclosure
Fear of negative reaction	Accusations of deception, physical and emotional abuse, being thrown out of relationships	Fear of violence and social consequences	
Healthcare workers are not providing disclosure guidance	No structured guidance on disclosure, lack of counseling on stigma navigation, unpreparedness for negative reactions	Lack of institutional support	
Selective disclosure to trusted individuals	Choosing trusted individuals to disclose to, avoiding disclosure to judgmental family members, balancing support-seeking with privacy	Psychosocial coping strategies	Strategies to facilitate PrEP disclosure
Peer support for PrEP adherence	Encouragement from peers to continue PrEP, reminders for medication adherence, and creating safe spaces for PrEP users	Social support strategies	
Healthcare provider support for disclosure	Confidential counseling and guidance, health workers clarifying PrEP misconceptions, using healthcare settings to support disclosure	Instrumental strategies	

Two researchers independently coded a randomly selected 20% of the transcripts to ensure reliability. Intercoder reliability was calculated and yielded a score of 0.81 (44). Discrepancies were resolved through discussion and consensus. Once reliability was established, the full data set was coded using the finalized codebook. Findings are presented with representative quotes from participants to illustrate the identified themes.

### Ethical approval

Ethical clearance was obtained from the Makerere University School of Social Sciences Research Ethics Committee (MAKSS-REC2019-299) and the Ministry of Health, AIDS Control Program (ADM.105/269/01). The MARPI clinic provided additional administrative clearance. RAs were experienced Ugandan healthcare workers with extensive work experience working with most at-risk populations. Participants were fully informed about the purpose of the study, study benefits and associated risks, their right to withdraw at any time, and the measures in place to ensure confidentiality. All identifying information was removed during transcription, and participants were provided access to support services in case of emotional distress during or after the interviews.

## Results

The study involved 14 Ugandan adolescent girls and young aged 18–24 years, all of whom were engaged in transactional sex and had experience with PrEP use. The participants had been on PrEP for varying durations, ranging from 1 to 5 years. Eleven (11) participants reported employing a selective disclosure approach to disclose their PrEP use to at least a family member, friend, or partner. Three (03) participants remained hesitant due to fears of stigma and judgment. The sample included individuals from diverse backgrounds, with varying levels of family support and exposure to healthcare services. Participants' narratives highlighted a wide range of experiences with PrEP disclosure, offering rich insights into the barriers faced and the strategies employed to navigate these challenges.

### Barriers to PrEP disclosure

The analysis of participants' narratives identified three key themes that encapsulate the primary barriers to PrEP disclosure: stigma and misconceptions about PrEP, fear of negative social and relational consequences, and lack of institutional and structural support.

### Theme 1: stigma, discrimination, and misconceptions

Stigma and discrimination were significant barriers to PrEP disclosure. Many participants experienced harmful assumptions that PrEP was the same as antiretroviral therapy (ARVs), leading others to believe they were HIV positive. These misconceptions fueled gossip, discrimination, stereotyping, and prejudice, forcing many to conceal their PrEP use to avoid judgment and exclusion. A significant challenge was the difficulty in convincing others that PrEP is for HIV prevention rather than treatment. Even when participants tried to explain, they were met with doubt and skepticism, as described by one participant:

“She said to me that the drugs looked exactly like ARVs. I gave her the package to read for herself to confirm that they are not ARVs. But she doubted me and said there was no way she would confirm that these weren't ARVs.” (Participant #011, 24 years old, Disclosed PrEP use, 3 years on PrEP).

False rumors about PrEP use often spread quickly, damaging reputations and leading to rejection from social circles:

“You tell someone that you are on PrEP but they will not believe you. They continue spreading gossip that you are on ARVs. That you are suffering from AIDS. That is the main reason why I am very selective with whom I reveal to that I am on PrEP.” (Participant #011, 24 years old, Disclosed PrEP use, 3 years on PrEP).

Some participants reported losing professional and personal opportunities due to stigma surrounding PrEP use:

“When people believe that you are on the ARVs, they begin rejecting you. They might even start refusing to share meals with you claiming that you will infect them with AIDS, which is not even possible. There are also some jobs where they want to know your health status. There could arise an opportunity which is useful to me but I would miss on such an opportunity because of the false belief that I have HIV.” (Participant #011, 24 years old, Disclosed PrEP use, 3 years on PrEP).

The strong association between PrEP and sex work exacerbated the stigma, leading to further social exclusion:

“I accidentally revealed that I was enrolled on PrEP (smiling). He told me one thing. That I am a prostitute. No woman uses PrEP unless she is a prostitute!…People talked a lot about me for using PrEP. My girlfriends, male friends and even family all called me a prostitute.” (Participant #001, 22 years old, Disclosed PrEP use, 4 years on PrEP).

For some, these experiences led to relationship breakdowns, reinforcing the difficulty of disclosure:

“My potential marriage partner broke up with me… He picked up the container and inquired about the drugs. He was informed that those were ARVs, and I was sick” (Participant #003, 23 years old, Disclosed PrEP use, 5 years on PrEP).

Even within families, PrEP users feared being misunderstood, leading them to hide their medication to avoid judgment:

“I have never told any family members. I wanted to tell my mom about it but feared that she would think that I was HIV positive but didn't want to admit it…I thought that they would assume that I was HIV positive but was lying to them. Trying to dilly-dally. I also feared being stigmatized. Being shunned that I was sick.” (Participant #008, 20 years old, Disclosed PrEP use, 2 years on PrEP).

For some individuals, the persistent stigma made them consider stopping PrEP altogether:

“Yes, they can. You could say, let me quit PrEP so that I am no longer discriminated or judged.” (Participant #008, 20 years old, Disclosed PrEP use, 2 years on PrEP).

## Theme 2: fear of violence and social repercussions

Fear of violence and relationship social repercussions emerged as another critical barrier to PrEP disclosure, particularly among adolescent girls and young women engaged in transactional sex. Participants reported facing physical aggression, emotional abuse, and social alienation when their partners, family members, or community members misunderstood the purpose of PrEP. These misunderstandings often led to accusations of deception, infidelity, or intent to transmit HIV, triggering violent confrontations and PrEP non-disclosure.

Several participants described how PrEP disclosure led to immediate accusations and the breakdown of their relationships:

“He then accused me of having intentions of infecting him with HIV. When I asked him why he made that accusation, he demanded to know why I was carrying those tablets. When I explained, he refused to believe me. He walked out on me without waiting for further explanation. Since then, I decided to leave them at home.” (Participant #001, 22 years old, Disclosed PrEP use, 4 years on PrEP).

For some, disclosure resulted in long-term emotional consequences, including being ignored and abandoned by their partners:

“He asked me one question. ‘All this long, you've been infected with HIV? Do you want to kill me?’ That phrase is used a lot. I have no idea as to why. He walked away. It was long without any communication between us. For several weeks, he wouldn't pick my calls.” (Participant #001, 22 years old, Disclosed PrEP use, 4 years on PrEP).

Beyond emotional distress, some individuals experienced coercive control from their partners, particularly those who were HIV positive and feared being left:

“He is aware. He was very pissed when I revealed it to him. He wanted me to also be infected with the virus like he is. He is fearful that I will quit the relationship. He always asks me why I swallow them. We normally quarrel about it.” (Participant #03, 23 years old, Disclosed PrEP use, 5 years on PrEP)

Physical violence was another direct consequence of PrEP disclosure. Some individuals reported being physically attacked by their partners, who assumed they were hiding an HIV-positive status:

“I had a boyfriend who landed on the PrEP tin in the house. He quarreled! He said, ‘You woman, all along you want to kill me? You are enrolled on ARVs?’ He was mad at me. He threw me out of the house. He became physical. We fought and attracted a crowd. He shouted and called to whoever cared to come and see the woman who wants to kill him.” (Participant #004, 24 years old, Disclosed PrEP use, 5 years on PrEP).

Beyond romantic relationships, family members also reacted violently upon learning about PrEP use. One woman feared disclosing to her mother due to concerns about her temper:

“I feared to disclose to her because sometimes she can easily lose temper and she abuses me.” (Participant #012, 23 years old, Disclosed PrEP use, 3 years on PrEP)

Another participant described being physically forced to visit a health facility for verification of her PrEP use:

“The first person I disclosed to wanted to beat me, just like my father. It wasn't easy as you can realize. They forcefully grabbed me, and we went to the health facility until they got confirmation from the health worker.” (Participant #09, 24 years old, Disclosed PrEP use, 2 years on PrEP)

Fear of violence extended beyond intimate and familial relationships to community spaces. Many participants worried about accidental discovery leading to rumors, exclusion, or physical harm:

“There could also be violence. You could be on PrEP in privacy, then a friend accidentally discovers it. Rumor can then spread that you are on ARVs and infected with HIV. You could therefore be threatened with violence, forcing you to shut up. You could be barred from participating in family forums because ‘you are infected’ or sharing common spaces. It could lead to depression and family divisions.”

## Theme 3: lack of institutional and structural support

Participants also cited gaps in healthcare and public health education as significant barriers to PrEP disclosure. Many individuals in their communities remained uninformed about PrEP, contributing to continued stigma and discrimination. The absence of targeted education campaigns made it difficult for AGYW to correct misconceptions when questioned about their PrEP use.

“People in my community don't know what PrEP is. If there were more awareness, maybe they wouldn't assume we are taking ARVs. Right now, if you try to explain, they just don't believe you.” (Participant #015, 21 years old, Disclosed PrEP use, 1 year on PrEP).

Beyond community awareness, participants noted that healthcare providers often did not offer adequate guidance on how to approach disclosure conversations. Many felt that structured counseling on PrEP communication would have helped them navigate stigma and misconceptions.

“When I first got PrEP, no one told me how to talk about it with my family or friends. I had to figure it out on my own. If health workers explained it better, it would be easier to disclose.” (Participant #016, 23 years old, Disclosed PrEP use, 2 years on PrEP).

The lack of structured community engagement was also highlighted as a major barrier. Participants believed that more advocacy through mass media, local meetings, and health campaigns would help normalize PrEP and reduce the burden of disclosure.

“They should talk about PrEP on the radio and in community meetings. Right now, people don't trust what they don't understand, and that's why they judge us.” (Participant #017, 22 years old, Disclosed PrEP use, 3 years on PrEP).

In addition to public awareness gaps, participants raised concerns about healthcare system barriers, including lack of privacy and confidentiality at health facilities. Many felt uncomfortable discussing PrEP in public spaces within clinics, which further deterred them from seeking support.

“Some clinics don't even have a private space to discuss PrEP. You have to talk about it in front of other patients, and that makes people uncomfortable. It's like they don't think about how hard it is for us to explain why we take it.” (Participant #018, 24 years old, Disclosed PrEP use, 4 years on PrEP).

Additionally, limited access to youth-friendly healthcare services left many participants without the necessary support to navigate PrEP disclosure. The lack of healthcare provider training on stigma reduction and PrEP counseling meant that participants received little to no guidance on how to communicate about their PrEP use safely.

“I wish the health workers understood what we go through. Sometimes, when you ask them about PrEP, they don't explain well, or they assume you already know everything. It makes it hard to open up or ask questions.” (Participant #019, 20 years old, Disclosed PrEP use, 2 years on PrEP).

## Strategies used to overcome barriers to PrEP disclosure

Through the lens of resilience, positive variation, and asset-based frameworks, the data illustrated how adolescent girls utilized various strategies across multiple social-ecological levels to navigate the complexities of PrEP disclosure. At the individual level, they drew on their personal strengths such as inner determination and self-confidence (resilience), in managing internal fears and stigma. At the interpersonal level, they leaned on peer networks and healthcare support systems (assets) to create trusted spaces for disclosure. At the community level, participants adapted their behaviors to different contexts or relationships (positive variation) to align with the social norms and expectations of different contexts and relationships. These adaptive strategies helped them overcome social and structural barriers, demonstrating how participants actively shaped their environments to foster safer spaces for disclosure across the individual, interpersonal, and community spheres.

## Theme 1: psychosocial coping strategies

Many participants relied on personal strength and self-motivation as key psychological strategies to navigate the challenges of PrEP disclosure. Self-reliance was often mentioned as a way to overcome societal judgment and internal fears:

“I am also a very strong person. Life is wealth. So, I am determined since I enrolled. I don't mind about other people's opinions because it's my life” (Participant #009, 24 years old, Disclosed, 2 years on PrEP).

Participants also emphasized the importance of self-belief and resilience, viewing these qualities as essential to overcoming the stigma and fear surrounding PrEP:

“They should just believe in themselves. Believe in themselves. They shouldn't fear to be judged” (Participant #007, 20 years old, Disclosed PrEP use, 1 year on PrEP).

Another participant shared how maintaining inner strength enabled her to continue using PrEP despite others' negative perceptions:

“This is my life. It's me who is at risk. If I quit PrEP and get infected with the HIV virus, they would still blame me for not enrolling on PrEP. So, I have to make my own decisions” (Participant #002, 20 years old, Disclosed PrEP use, 2 years on PrEP).

Several participants practiced selective disclosure, revealing PrEP use only to those they trusted deeply, which helped them manage the psychological burden of potential stigma. By disclosing only to close friends, participants reduced the risk of gossip or judgment:

“Friends can even spoil my name where I stay. Unless you are a very close friend. The one I disclosed to is a very close friend” (Participant #011, 24 years old, Disclosed PrEP use, 3 years on PrEP).

Selective disclosure was often a method of self-protection, allowing participants to balance the need for support with the desire to avoid adverse reactions from their social circles:

“I am not very close to family. They don't know much about me. It's impossible for me to discuss it with family. There's no one I am close to… I don't want to cause a heart attack to my old mother” (Participant #003, 23 years old, Disclosed PrEP use, 5 years on PrEP).

## Theme 2: social support coping strategies

Leveraging peer support was one of the most common social strategies for PrEP disclosure. Many participants revealed that disclosing PrEP use to close friends or fellow sex workers provided emotional support, helped them manage adherence, and allowed them to normalize PrEP within their social circles. For example, one participant reported:

“My friend whom I disclosed to always inquires as to whether I am adhering to the drugs. She also reminds me when we should go and pick more medicine. She also asks me about any possible side effects” (Participant #011, 24 years, Disclosed PrEP use, 3 years on PrEP).

Peers also provided crucial emotional and logistical support, encouraging one another to adhere to their medication and reminding each other of its importance:

“We always encourage each other. For example, if the date has reached for picking the medicine, we remind each other. We accompany each other to go and collect our medicine” (Participant #012, 23 years old, Disclosed PrEP use, 3 years on PrEP).

Participants shared how friends played a vital role in reassurance and helping them stay on track with their medication:

“She [my friend] also reassures me that the drug is effective, asking me whether I swallow the drugs properly” (Participant #011, 24 years old, Disclosed, 3 years on PrEP).

This peer support often extended into practical help, with some participants acting as informal leaders among their social group, guiding others through the process of PrEP disclosure and adherence:

“With my fellow sex workers, yes. Some of them I helped to enroll request me to obtain the drugs for them and refer to me as their leader. They trust and consult me if they have concerns about PrEP” (Participant #002, 20 years old, Disclosed PrEP use, 2 years on PrEP).

Although family support was less frequently mentioned, some participants disclosed PrEP use to trusted family members, particularly mothers or siblings. This selective disclosure helped alleviate anxiety, with participants feeling comforted by their family's acceptance:

“Maybe my mother whom I also disclosed to because she cannot betray me. She told me that if it gives me protection, then I don't have to miss it” (Participant #012, 23 years old, Disclosed PrEP use, 3 years on PrEP).

## Theme 3: instrumental coping strategies

Evidence-based healthcare provider support played a crucial role in facilitating PrEP disclosure among adolescent girls engaged in transactional sex in Uganda***.*** Healthcare providers played an instrumental role in encouraging disclosure and adherence by offering confidential, evidence-based guidance. These interactions provided participants with the confidence needed to manage both their medication and the social aspects of disclosure:

“It is easy for me to talk to you about myself because you medical workers have a rule which says that whatever we discuss here is confidential” (Participant #011, 24 years old, Disclosed PrEP use, 3 years on PrEP).

Healthcare workers often served as trusted sources of information, offering reassurance when participants faced side effects or doubts about PrEP:

“They told me it was a normal reaction when one has just enrolled on PrEP to experience headache. I was advised to drink a lot of fluids. They can also help you on how you go around with telling others about PrEP use” (Participant #001, 22 years old, Disclosed PrEP use, 4 years on PrEP).

In addition to individualized support, healthcare providers were instrumental in facilitating broader community education about PrEP. They helped participants deflect the burden of convincing others by directing them to professionals for further explanation:

“They should urge me that if my colleagues do not believe in me, I should mobilize them to come to the clinic for further information so that they can also enroll” (Participant #010, 23 years old, Disclosed PrEP use, 2 years on PrEP).

Some participants even relied on health workers to help explain PrEP use to skeptical family members or friends, allowing them to disclose without carrying the full responsibility of convincing others:

“I took my mother to the health worker who clarified to her that I was on PrEP and not ARVs” (Participant #009, 24 years old, Disclosed PrEP, 2 years on PrEP).

## Theme 4: multiple strategies

Most participants employed multiple strategies to navigate the complexities of PrEP disclosure. These strategies often included a combination of social, psychological, and technical/instrumental approaches. Participants demonstrated a range of adaptive behaviors by drawing on their peer networks, healthcare providers, and personal resilience simultaneously. By leveraging multiple resources, they were able to mitigate the barriers of stigma, judgment, and fear of violence. For example, one participant described how she combined peer support with self-motivation and healthcare provider guidance to overcome her fear of judgment and stay committed to PrEP adherence:

“I have support from my friends without whom I would no longer be on PrEP. It's the main reason I have the guts to disclose because the entire group is enrolled… so, I also encourage others to enroll. My health worker also told me it's the best option I have to protect myself” (Participant #009, 24 years old, Disclosed PrEP use, 2 years on PrEP).

Another participant shared how she used both peer support and healthcare providers as her primary strategies, allowing her to manage both the emotional and practical challenges of disclosure:

“My friend whom I disclosed to always inquires as to whether I am adhering to the drugs. She also reassures me that the drug is effective. I also rely on my healthcare worker, who guides me and gives me information that I use when I explain PrEP to others” (Participant #011, 24 years old, Disclosed PrEP use, 3 years on PrEP).

## Discussion

Participants in this study faced significant barriers to PrEP disclosure across multiple levels of the social-ecological model, including individual (stigma, misconceptions about PrEP, and fear of judgment), interpersonal (fear of rejection, relationship breakdown, and violence from partners, clients, and family members), community (stigma linking PrEP to HIV treatment and sex work) and structural (misinformation and inadequate public health education about PrEP). Among these barriers, stigma emerged as a particularly pervasive barrier to disclosure among almost all participants. At the individual level**,** participants grappled with personal fears of judgment and anxiety over being mislabeled as HIV-positive due to the physical resemblance of PrEP pills to antiretroviral therapy (ARV) medication. This often led to non-disclosure, even to close family members, as participants feared rejection and being perceived as promiscuous or untrustworthy. At the community level, participants reported PrEP non-disclosure due to fear of stigma and being judged by others in the communities. Studies with at-risk women and sex workers frequently show that stigma from the community significantly affects willingness to continue using PrEP (45, 46).

Stigma was deeply entrenched in inadequate information related to PrEP leading to misconceptions that equate PrEP with HIV treatment and sex work, resulting in social rejection and discrimination. These misconceptions about PrEP often led individuals to conceal PrEP use or selectively disclose it only to trusted persons (20, 47, 48). This issue was particularly pronounced among younger girls, who, due to low education levels, has inadequate access to accurate information and were more susceptible to misinformation about PrEP compared to their older counterparts. These findings are consistent with prior research in Sub-Saharan Africa, where HIV-related stigma is attributed to misinformation and are key barriers to PrEP uptake (49) and disclosure (20). Improving PrEP awareness is critical in efforts to increase PrEP disclosure among AGYW who engage in transactional work in Uganda (50). PrEP awareness campaigns should not only be geared towards widespread awareness but should employ youth-friendly channels popular with young people, such as TikTok, WhatsApp, Instagram, and Facebook, and should have targeted relevant messages that meet the needs of young people to capture their attention.

Participants employed various coping strategies, including selective disclosure, peer support, and healthcare provider guidance, to navigate PrEP disclosure challenges, reduce stigma, and maintain adherence. Strength-based approaches could help bolster participants coping strategies by addressing misconceptions around PrEP. One such approach may be mass sensitization campaigns and community-engaged solutions that emphasize that PrEP is a preventive intervention and not a treatment. This strategy could help normalize PrEP use and foster safer environments for disclosure, aligning with evidence from studies (51) that stress the role of informed and supportive communities in empowering adolescent girls engaged in sex work to navigate disclosure without fear of judgment or violence.

Our study highlighted the crucial role of healthcare provider support in helping adolescent girls engaged in sex work navigate disclosure challenges, which aligns with previous research from Uganda (48, 52, 53). Participants who had strong, trusting relationships with healthcare providers were more likely to disclose PrEP use, as these providers offered guidance, reassurance, and practical solutions to alleviate fears of judgment or violence. Consistent with findings by Kiwanuka et al. (59), healthcare providers acted as trusted sources of support, reinforcing the importance of a patient-centered approach in PrEP programs. Implementation strategies should focus on training providers to use techniques such as motivational interviewing and confidentiality assurances to improve both adherence and disclosure outcomes, as suggested by similar studies on HIV prevention (14). Furthermore, healthcare providers can play a critical role in educating families and partners about PrEP, reducing misconceptions, and supporting disclosure, as many participants in this study relied on health workers to mediate discussions with skeptical family members.

The use of multiple strategies—including peer support, selective disclosure, and self-motivation—underscores the need for implementation strategies that take a comprehensive, holistic approach. Participants in this study combined social strategies with psychological resilience and technical support from peers, adherence supporters (54), and healthcare providers, reflecting findings from previous studies in Uganda (55). These studies have similarly suggested that no single approach is sufficient to overcome the complex barriers to PrEP disclosure, especially among adolescent girls engaged in sex work. Multi-level interventions that address individual, interpersonal, and systemic factors are essential, as supported by Mugwanya et al. (56), who advocate for integrated models of care. For instance, peer-led PrEP support groups, as mentioned by Wamoyi et al. (57), could be incorporated into existing healthcare structures to provide both emotional support and practical guidance for these adolescents. Furthermore, implementation strategies should ensure that PrEP services are connected to broader social support systems, including mental health services, to help alleviate the psychological burden of stigma and judgment.

## Implications for policy and practice

The findings of this study have significant implications for both policy and practice in HIV prevention for adolescent girls engaged in transactional sex in Uganda. Reducing stigma and misconceptions around PrEP must be a key priority, and this can be achieved through targeted community-level education campaigns that clearly communicate PrEP's preventive purpose. Policymakers should consider leveraging mass media, peer educators, and community sensitization events to shift public perceptions. Furthermore, healthcare providers play an essential role in fostering an environment where PrEP users feel supported. Training healthcare workers in patient-centered approaches, including maintaining confidentiality and using motivational interviewing, is critical for enhancing PrEP adherence and facilitating safer disclosure.

Additionally, implementing multi-level interventions that integrate peer support groups with healthcare services could offer both emotional and practical support to those navigating PrEP use and disclosure. These programs should be paired with mental health and trauma-informed services to address the psychological challenges, stigma, and violence faced by participants. Policymakers should focus on scaling up access to PrEP by offering low-barrier services, such as mobile clinics, to reach this vulnerable population. Collaborative efforts between public health officials, community leaders, and NGOs will be crucial in ensuring that these interventions reach the most at-risk individuals, providing them with the tools to protect their health.

## Limitations and future research

This study is not without limitations. Firstly, the research focused exclusively on adolescent girls engaged in transactional sex in Uganda, which may limit the generalizability of the findings to other key populations or regions. The reliance on self-reported data also introduces potential biases, such as social desirability bias, where participants may have underreported or overreported their experiences with PrEP use and disclosure. Additionally, the study did not capture the perspectives of male partners, healthcare providers, or other stakeholders who could provide complementary insights into the challenges of PrEP disclosure. Moreover, the use of secondary data collected before the COVID-19 pandemic means that it provides only a snapshot of participants' experiences, potentially overlooking the evolving nature of stigma, support systems, and PrEP use over time.

Future research should focus on longitudinal studies to track the changing dynamics of PrEP use and disclosure among adolescent girls in similar settings. Including diverse perspectives, such as those of partners, family members, and healthcare providers, would provide a more comprehensive understanding of the factors that influence PrEP disclosure. Further research is also needed to explore the effectiveness of multi-level interventions that integrate mental health, peer support, and healthcare services in improving PrEP outcomes. Finally, future studies should examine the intersection of PrEP use with other social determinants of health, such as economic status, education, and access to healthcare, to better tailor interventions for this vulnerable population.

## Conclusion

This study highlights the complex strategies adolescent girls engaged in transactional sex use to navigate PrEP disclosure amidst significant barriers such as stigma, discrimination, and violence. By drawing on resilience, positive variation, and asset-based approaches, participants were able to overcome these challenges and maintain adherence to protect themselves from HIV infections. These findings underscore the need for targeted interventions that combine community education, healthcare support, and peer-led programs to address both the barriers to disclosure and the adaptive behaviors already present in these communities. Empowering vulnerable populations through a focus on resilience and community assets can enhance PrEP disclosure and adherence, ultimately improving HIV prevention outcomes.

## Data Availability

The raw data supporting the conclusions of this article will be made available by the authors, without undue reservation.
