# Supplementary material for: Identifying barriers and coping strategies for pre-exposure prophylaxis disclosure: experiences of Ugandan adolescent girls engaged in transactional sex—a qualitative study
Source: Front Glob Womens Health. 2025 Mar 20;6:1517448. doi: 10.3389/fgwh.2025.1517448 (PMC11965671; doi:10.3389/fgwh.2025.1517448)
Supplement: Supplementary file 1 [file Table1.pdf]

Supplementary File 1: COREQ checklist

Consolidated criteria for reporting qualitative studies (COREQ): 32-item checklist

Developed from:
Tong A, Sainsbury P, Craig J. Consolidated criteria for reporting qualitative research (COREQ): a 32-item checklist for interviews and focus groups. International Journal for Quality in Health Care. 2007. Volume 19, Number 6: pp. 349 – 357

Item No	Guide Questions/Description	Reported on Page #/Section
**Domain 1: Research team and reflexivity**		
**Personal Characteristics**		
1. Interviewer/ facilitator	Which author/s conducted the interview or focus group?	Pg 04 (Data collection section)
2. Credentials	What were the researcher's credentials? E.g., PhD, MD	Pg 04 (Data collection section)
3. Occupation	What was their occupation at the time of the study?	Pg 04 (Data collection section)
4. Gender	Was the researcher male or female?	Pg 04 (Data collection section)
5. Experience and training	What experience or training did the researcher have?	Pg 04 (Data collection section)
**Relationship with participants**		
6. Relationship established	Was a relationship established prior to study commencement?	Pg 04 (Recruitment section)
7. Participant knowledge of the interviewer	What did the participants know about the researcher? e.g. personal goals, reasons for doing the research?	Pg 04 *(Data collection section*)
8. Interviewer characteristics	What characteristics were reported about the interviewer/facilitator? e.g. Bias, assumptions, reasons and interests in the research topic	Pg 04 (Data collection section)
**Domain 2: study design**		
**Theoretical framework**		

Item No	Guide Questions/Description	Reported on Page #/Section
9. Methodological orientation and Theory	What methodological orientation was stated to underpin the study? e.g. grounded theory, discourse analysis, ethnography, phenomenology, content analysis	Pg 02-03 (Theoretical Frameworks section)
**Participant selection**		
10. Sampling	How were participants selected? e.g., purposive, convenience, consecutive, snowball	Pg 04 (Methods, recruitment section)
11. Method of approach	How were participants approached? e.g., face-to-face, telephone, mail, email	Pg 04 (Methods, recruitment section)
12. Sample size	How many participants were in the study?	Pg 04 (Methods, recruitment section)
13. Non-participation Setting	How many people refused to participate or dropped out? Reasons?	Pg 04 (Methods, recruitment section)
14. Setting of data collection	Where was the data collected? e.g., home, clinic, workplace	Pg 04 (Data collection, participants section)
15. Presence of nonparticipants	Was anyone else present besides the participants and researchers?	N/A
16. Description of sample	What are the important characteristics of the sample? e.g. demographic data, date	Pg 04 (Data collection, participants section)
**Data collection**		
17. Interview guide	Were questions, prompts, and guides provided by the authors? Was it pilot tested?	Pg 04 (Data collection, participants section)
18. Repeat interviews	Were repeat interviews carried out? If yes, how many?	N/A
19. Audio/visual recording	Did the research use audio or visual recording to collect the data?	Pg 04 (Data collection, data analysis section)

Item No	Guide Questions/Description	Reported on Page #/Section
20. Field notes	Were field notes made during and/or after the interview or focus group?	Pg 04 (Data collection, participants section)
21. Duration	What was the duration of the interviews or focus group?	Pg 04 (Data collection, participants section)
22. Data saturation	Was data saturation discussed?	Pg 04 (Data collection, participants section)
23. Transcripts returned	Were transcripts returned to participants for comment and/or correction?	N/A
**Domain 3: analysis and findings**		
**Data analysis**		
24. Number of data coders	How many data coders coded the data?	Pg 04 (Data collection, data analysis section)
25. Description of the coding tree	Did the authors provide a description of the coding tree?	N/A
26. Derivation of themes	Were themes identified in advance or derived from the data?	Pg 04 (Data collection, data analysis section)
27. Software	What software, if applicable, was used to manage the data?	Pg 04 (Data collection, data analysis section)
28. Participant checking	Did participants provide feedback on the findings?	N/A
**Reporting**		
29. Quotations presented	Were participant quotations presented to illustrate the themes/findings? Was each quotation identified? e.g., participant number	Pg 05-09 (Results section)
30. Data and findings consistent	Was there consistency between the data presented and the findings?	Pg 05-09 (Results section)
31. Clarity of major themes	Were major themes clearly presented in the findings?	Pg 05-09 (Results section)

Item No	Guide Questions/Description	Reported on Page #/Section
32. Clarity of minor themes	Is there a description of diverse cases or a discussion of minor themes?	Pg NA
